# Supplementary material for: Engineering Moderately Lithiophilic Paper-Based Current Collectors with Variable Solid Electrolyte Interface Films for Anode-Free Lithium Batteries
Source: Nanomaterials (Basel). 2024 Sep 8;14(17):1461. doi: 10.3390/nano14171461 (PMC11396866; doi:10.3390/nano14171461)
Supplement: Supplementary file 1 [file nanomaterials-14-01461-s001.zip › nanomaterials-3182088-supplementary.pdf]

Supporting information

Engineering Moderately Lithiophilic Paper-based Current Collectors with Variable Solid Electrolyte Interface Films for Anode-Free Lithium Batteries

*Baohong Yang[1,2,†], Hairu Wei[1,†], Huan Wang[1], Haoteng Wu[1], Yanbo Guo[1], Xuan Ren[1], Chuanyin Xiong, Hanbin Liu[1,2], Haiwei Wu[1,2]**

1 College of Bioresources Chemical & Materials Engineering, Shaanxi University of Science & Technology, Xi'an, Shaanxi 710021, China.

2 Shaanxi Provincial Key Laboratory of Papermaking Technology and Specialty Paper Development, Xi'an, Shaanxi 710021, China.

Dr. Haiwei Wu, Email: haiweiwufly@163.com, wuhaiwei@sust.edu.cn

[†]*Baohong Yang and Hairu Wei contributed equally to this work.*

The conductivity was measured using the four-probe method (RTS-9, Guangzhou Four Probe Technology Co., Ltd.). The sample was prepared as a 10 mm × 10 mm thin sheet and placed on the four-probe testing platform with a probe spacing of 1 mm. The conductivity, σ, was calculated using the formula $\sigma = I / (V * t)$, where t is the thickness of the sample. To minimize error, each sample was measured three times, and the average value was taken as the final result.

Table S1 Conductivity analysis of each composite current collector

Current Collector	Average Conductivity（S cm^{-1}）
LDP@Cu	1150
LDP@Cu-Ag	1280
LDP@Ag	1250

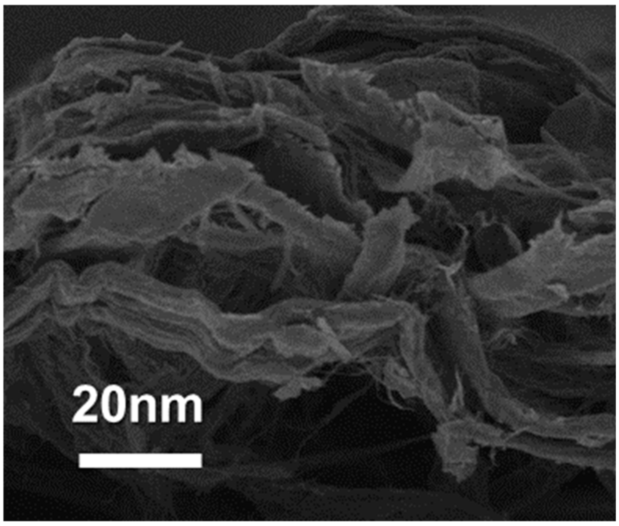

Fig.S1 SEM image of LDP

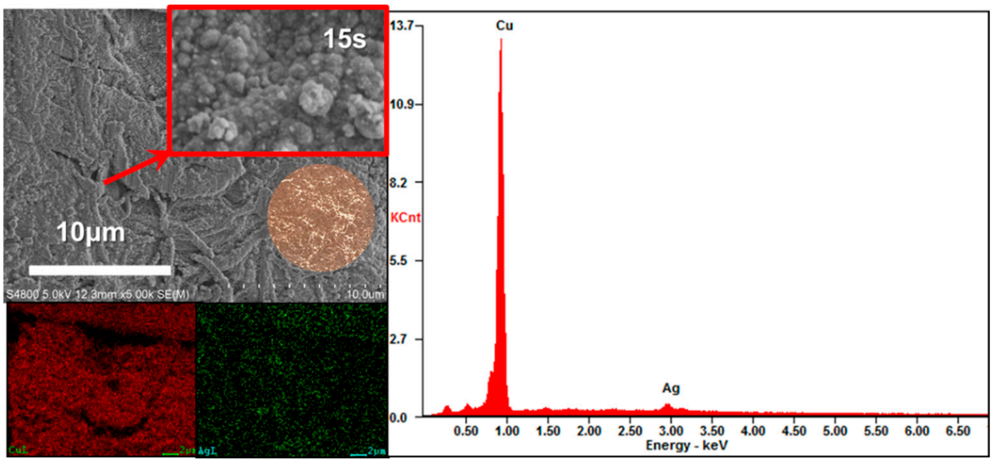

Fig. S2 EDS spectra of LDP@Cu-Ag

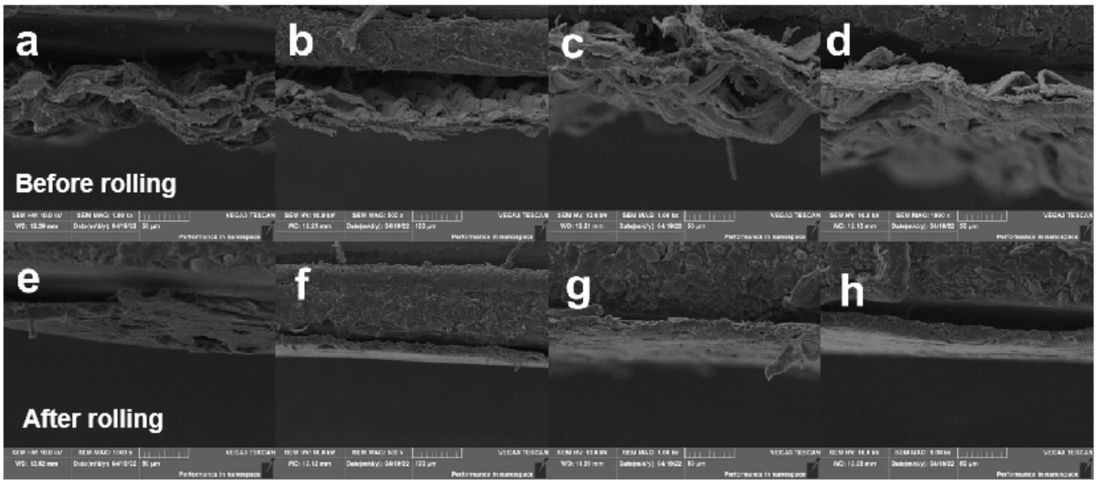

Fig. S3 SEM cross-section images of a,e) LDP, b,f) LDP@Cu, c,g) LDP@Cu-Ag, and d,h) LDP@Ag before and after rolling

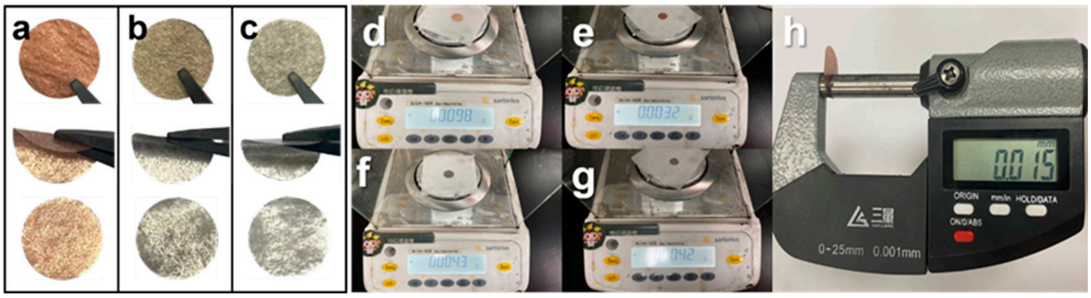

Fig.S4 Optical picture of each composite current collector. a) LDP@Cu b) LDP@Cu-Ag c) LDP@Ag. d) Commercial copper foil e) LDP@Cu f) LDP@Cu-Ag g) Mass of LDP@Ag and h) Thickness

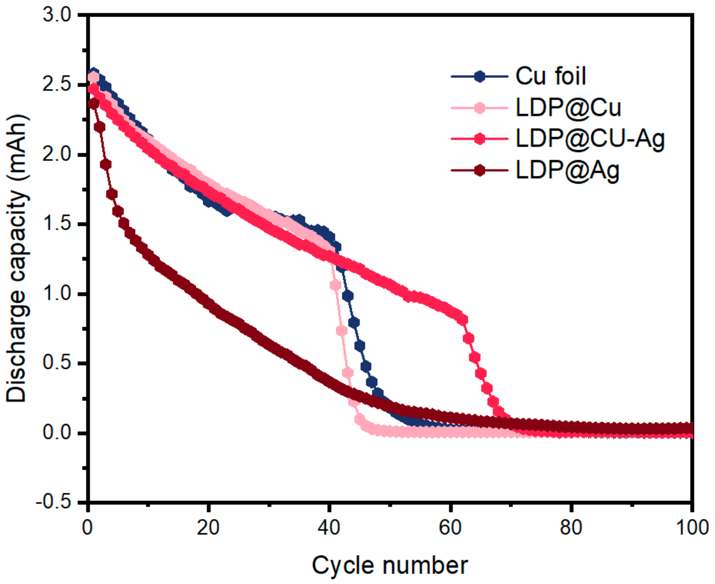

Fig. S5 LDP@XX||LFP with an ether-based electrolyte (1M LiTFSI DME-DOL) under 0.1C/0.3C charge/discharge rates

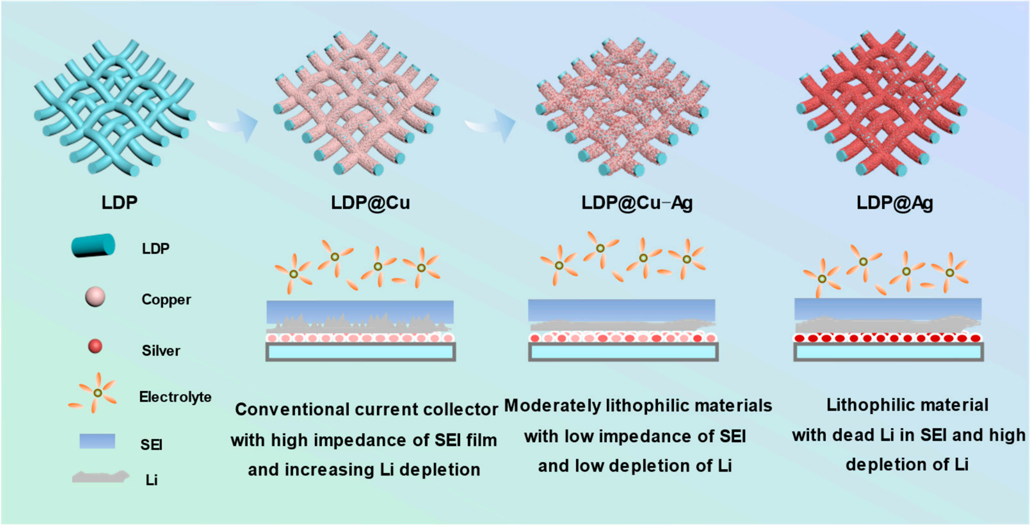

Fig.S6 Scheme of Li depletion in AFLMB with different lithophilic current collectors

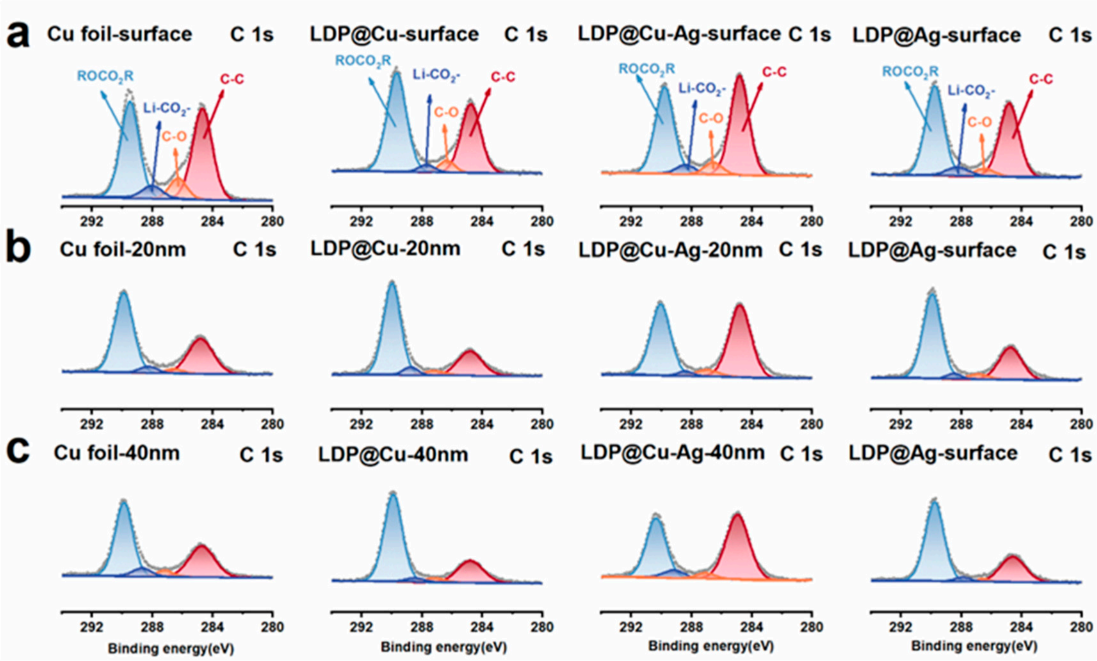

Fig. S7 Argon ion sputtering onto a）the surface b）at 20 nm c）at 40 nm for XPS C-1s spectra

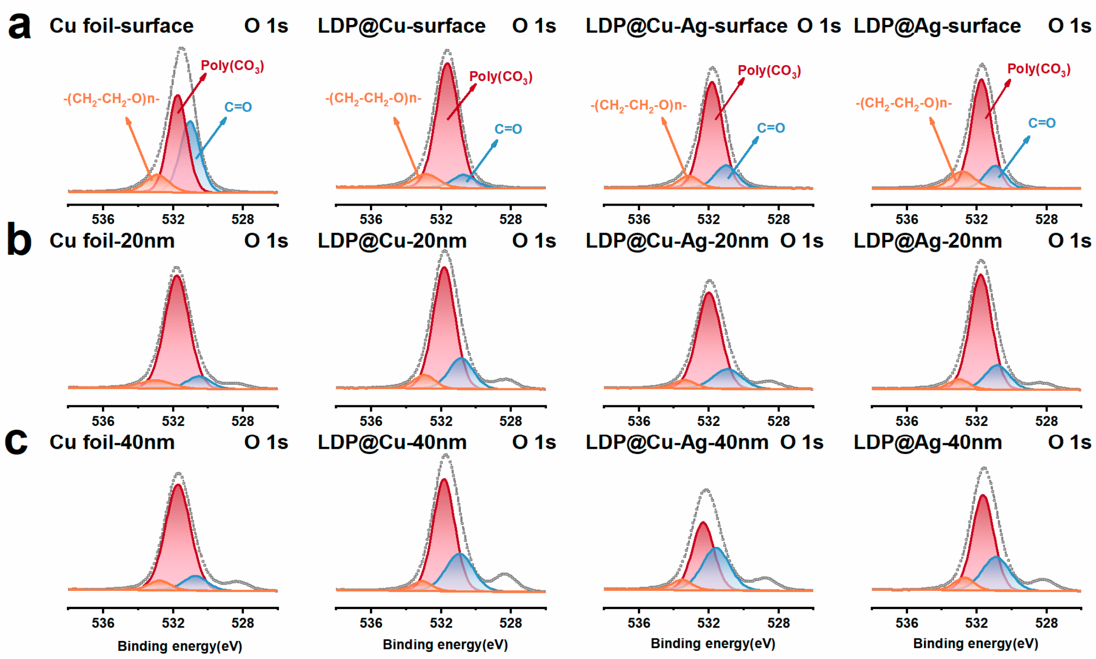

Fig. S8 Argon ion sputtering onto a）the surface b）at 20 nm c）at 40 nm for XPS O-1s spectra
